# A toolbox for automated driving on the STISIM driving simulator

**DOI:** 10.1016/j.mex.2018.08.003

**Published:** 2018-08-15

**Authors:** Alexander Eriksson, Joost de Winter, Neville A. Stanton

**Affiliations:** a Transportation Research Group, Faculty of Engineering and Environment, University of Southampton, Boldrewood Campus, Southampton SO16 7QF, UK; b Department of Biomechanical Engineering, Faculty of Mechanical, Maritime and Materials Engineering, Delft University of Technology, Delft, The Netherlands; c The Swedish National Road and Transport Research Institute, Box 8072, SE-402 78 Göteborg, Sweden

**Keywords:** Software toolbox, Driving simulator, Automated driving, Toolbox, Human factors, Adaptive cruise control, Highly automated driving, STISIM

## Abstract

Driving simulators have been used since the beginning of the 1930s to assist researchers in assessing driver behaviour without putting the driver in harm’s way. The current manuscript describes the implementation of a toolbox for automated driving research on the widely used STISIM platform. The toolbox presented in this manuscript allows researchers to conduct flexible research into automated driving, enabling independent use of longitudinal control, and a combination of longitudinal and lateral control, and is available as an open source download through GitHub. The toolbox allows the driver to adjust parameters such as set speed (in 5 kph increments) and time-headway (in steps of 1, 1.5, and 2 s) as well as automation mode dynamically, while logging additional variables that STISIM does not provide out-of-the-box (time-headway, time to collision). Moreover, the toolbox presented in this manuscript has gone through validation trials showing accurate speed, time-headway, and lane tracking, as well as transitions of control between manual and automated driving.

• A toolbox was developed for STISIM driving simulators.
• The toolbox allows for automated driving.
• Functionality includes tracking of speed, headway, and lane.

## Graphical abstract

**Figure:**
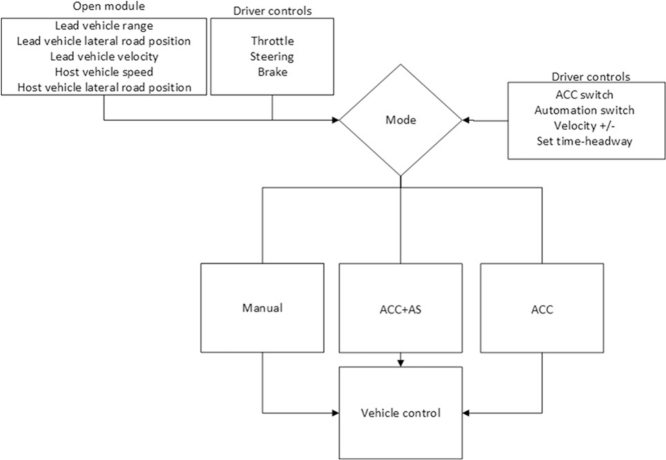

**Specifications Table**

**Table:** 

Subject Area	*Psychology*
More specific subject area	*Human Factors*
Method name	*Software toolbox*
Name and reference of original method	Not applicable, see paper
Resource availability	https://github.com/he1y13/Toolbox-for-automated-driving-in-STISIM

## Method description

In this paper, we describe a set of algorithms developed for the STISIM driving simulator platform. The goal of the algorithms was to enable dynamic human-automation interaction through custom software using the STISIM V3 Build 3.07.04 Open Module in Visual Basic 6 (VB6). Although this implementation of automated driving is platform-specific, the toolbox can be implemented on other platforms that offer an API or SDK by translating the subroutines into the programming language supported by the simulator in question, with the requirement that the lead-vehicle can be identified and queried for information such as speed through the API/SDK.

Open Module is a plugin feature of the STISIM platform that allows researchers to implement their modules using unmanaged code (e.g., VB6 or C++). One of the functions of Open Module is the ‘Update’-function which is called once every simulation frame, just before the graphics display updates. The ‘Update’-function allows the researcher to directly control the behaviour of a vehicle via pedal and steering input, a functionality that was utilised in developing our toolbox. The toolbox consists of several subroutines, each responsible for a part of the vehicle control, allowing lateral and longitudinal automation to be used separately or in conjunction. The functionality of the toolbox algorithms is detailed below.

## Algorithms

**Fig. 1:**
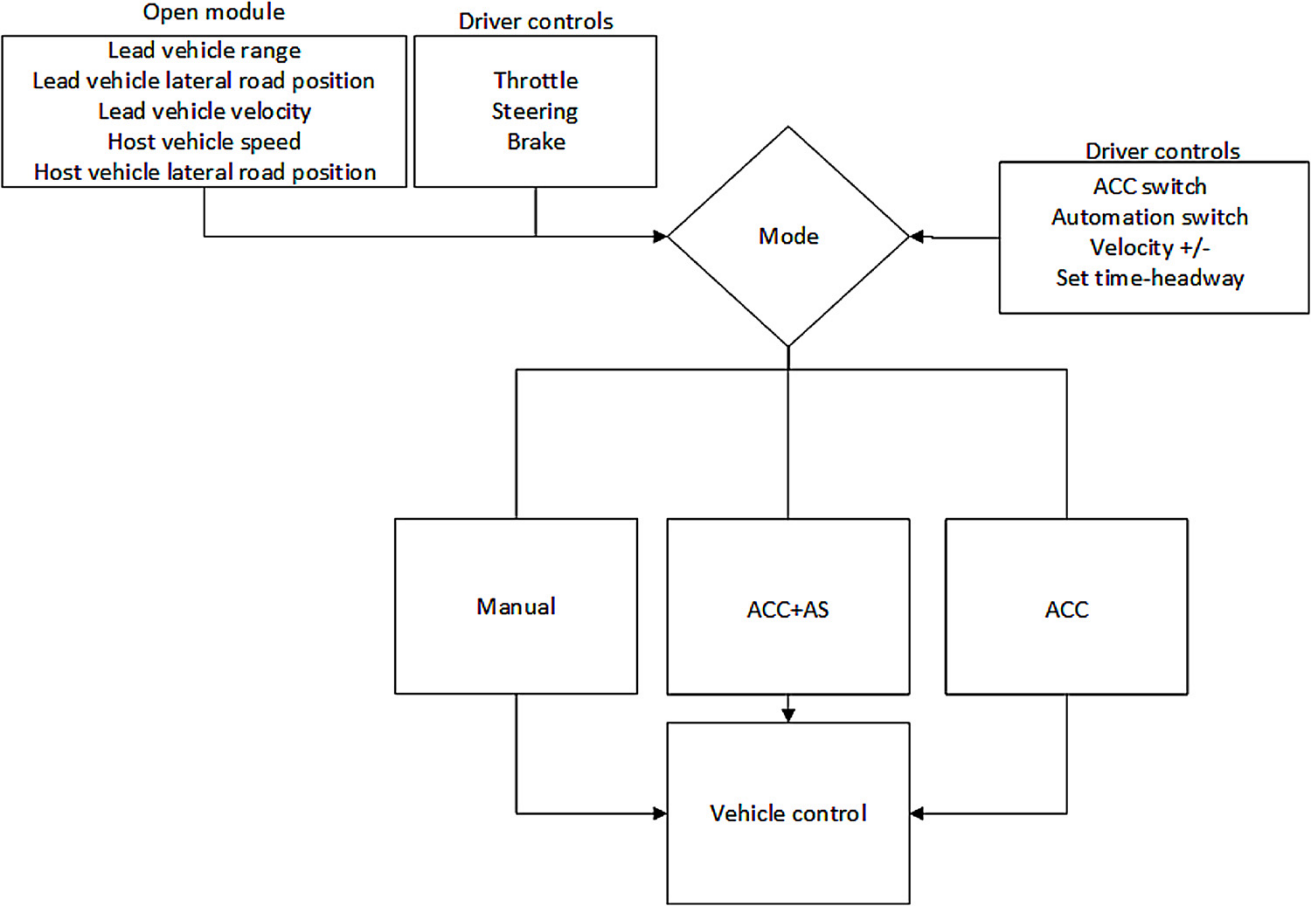
A functional block diagram of the automated driving toolbox. AS refers to automated steering control. Host vehicle refers to the vehicle equipped with the described algorithm.

In this section the algorithms are described in two parts: (1) longitudinal control and (2) lateral control. This structure enables the simulation of different levels of automated driving, ranging from manual driving and ACC (i.e., automated longitudinal control) to highly automated driving (i.e., automated longitudinal and lateral control) as shown in Fig. 1. The manual mode is void of any automated features, meaning that the operation of the vehicle is dependent on the human driver only.

**Fig. 2:**
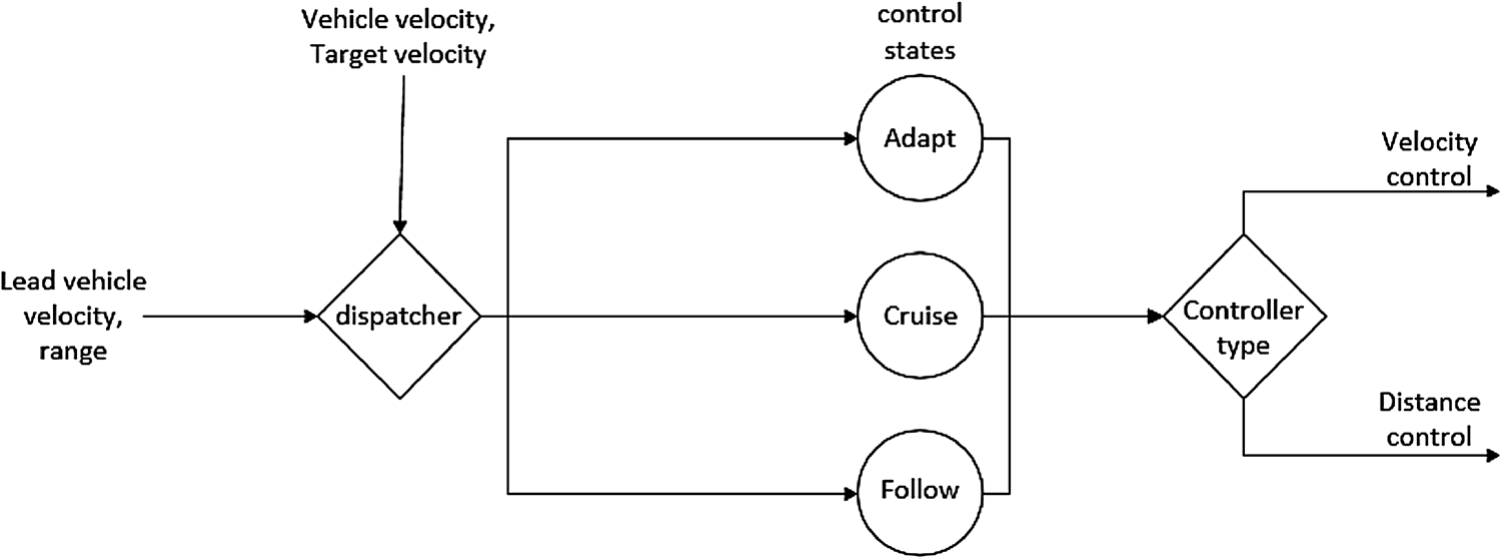
Functional block diagram of the longitudinal (ACC) control algorithm.

The ACC mode shown in Fig. 1 controls the vehicle’s speed by providing control signals to the throttle and brake inceptors to drive the vehicle at a target speed. This mode is broken down into its constituents in Fig. 2. The target speed is set by the driver or is dictated by the speed of a slower vehicle within sensor range (this range may be changed in the source code to simulate radars with higher or lower range). Additionally, there is an option to uncomment a section of the code in ‘OM_Module.cls’ which will set the maximum speed to that of the speed limit. ACC is an integral part of achieving highly automated driving and is now commonly available in production vehicles. ACC on production vehicles utilises a radar unit attached to the front of the vehicle that keeps track of any leading vehicles, feeding the cruise control algorithm with the distance to the lead vehicle, which is used to compute the desired speed to maintain the selected time-headway.

The highly automated driving mode incorporates the functionality of the ACC feature, with the addition of automated lateral control. The host vehicle (host vehicle refers to the vehicle equipped with the described algorithm) automatically follows the road curvature. In addition, lane changes may be performed in response to driver commands, by for example flicking the indicator stalk in the direction of the lane-change when in highly automated driving mode, much like a function of the most recent addition of automation on the market, the Tesla Motors [1] Autopilot Lane change functionality (however, in its current state the automation does not assess the traffic in the adjacent lane before changing lane). The longitudinal and lateral automation subroutines are further explained in the sections below.

### Longitudinal automation

#### Longitudinal automation functionality and states

The primary function and fundamental requirement of a longitudinal control system is to control and adapt speed to leading vehicles and driver settings (this is referred to as target speed, *v_target_*). Further requirements and assumptions for a longitudinal (i.e., ACC-based) control system are that:

1 the system cannot be engaged while the vehicle is driving in reverse [2].
2 the driver has the ability to override the system.
3 the system maintains the speed set by the driver in the absence of a slow leading vehicle.
4 the system uses acceleration thresholds to ensure comfortable driving during normal operating conditions.
5 the system slows down the vehicle to the speed of a slower moving lead vehicle and maintains the desired headway.
6 the system ignores deceleration thresholds (in terms of comfort) when such a threshold hinders bringing the vehicle to a safe system state through slowing down or stopping completely.
7 the system hands back control to the driver when the operational limits are approached. Such limits may include sensor failure, geographical constraints, or external factors leading to degraded system performance (this can be simulated through a shutdown event specified in an event file in the automation toolbox).

In the algorithm, the leading vehicle is considered to be a vehicle driving in the host vehicle’s lane (it is in the host vehicle lane when its centre of mass is within the lane boundary) within the range of the simulated radar. The controller works in accordance with SAE J2399 [3] on all points except that our controller does not allow for setting a minimum speed, and that it does not activate an auditory, visual, and/or haptic alert to inform the driver that he/she needs to take back manual control. The former lacks compliance because our controller works as a stop&go system. The latter limitation comes down to the design and implementation of a human-machine interface, which is not covered in the current manuscript. However, it is possible to add an interface by enabling the socket connection described elsewhere in this paper.

We believe that this controller for ACC works well enough for most Human Factors applications. However, normally, ACC systems on the market include more complex control algorithms for longitudinal control, such as gain scheduling proportional integral control (GSPI), gain scheduling linear quadratic control (GSLQC) [4] and nonlinear model predictive control (NMPC) [4]. The implementation of such control algorithms is straightforward: Should one wish to use a more advanced control algorithm, the controller used in the toolbox can be replaced with another type of control, either an implementation of one of the above mentioned or a vehicle manufacturer’s version. It must, however, be noted that Visual Basic 6 does not support multithreading, and thus, all the code in the Open Module are executed in the same thread as the simulation, so the addition of more complex functionalities may have an effect on the simulator performance (i.e., frame drops, lag etc.).

The algorithm to access vehicle data for the lead-vehicle is described in Eriksson and Stanton [5]. Parameters related to the lead-vehicle are denoted with the subscript *lead*. The longitudinal control algorithm is designed as a Finite State Machine (FSM), containing three states (cruise, follow, and adapt), each with its controller characteristics. Several conditions need to be fulfilled before the FSM can transition from one state to another. The process of determining controller states are shown in Pseudocode 1. The fixed parameters in the below pseudocode were tuned manually to ensure smooth switching between the control states of the FSM.

**Figure:**
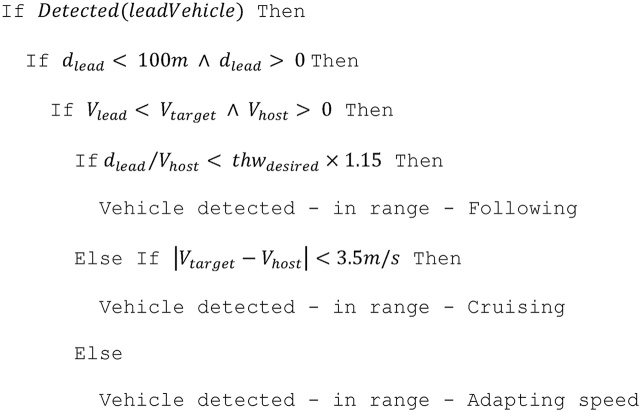



Pseudocode 1: the algorithm to determine the stage of the longitudinal control subroutine. Note: ∧ is the logical ‘AND’ operator. The 1.15 multiplier for the time-headway condition ensures that there is no sudden switching of time-headway and speed based error terms. ‘Too fast’ refers to the lead vehicle travelling faster than the desired speed set by the driver

Each state has a Proportional-Integral-Derivative (PID) controller and depending on the state, different gains for the different parameters are used. The transfer function of a PID controller is the sum of the outputs of three sub-controllers: a proportional, an integral, and a derivative controller. The error signal undergoes processing in each controller (i.e., the proportional, integral, and derivative sub-controllers), the resulting signals are added and constitute the total output from the PID controller.

In the case of automation, one of the inputs to the control system is the target speed, and the output is a number representing the virtual pedal position. Positive output values are signals sent to the virtual throttle pedal. For negative signals, their absolute values represent the virtual brake pedal position.

Because the environment is inherently digital, discrete mathematics applies to the computations. Hence, the temporal resolution (Δt) is limited to a single simulation frame, i.e., 1/30 s for a frequency of 30 Hz. The controller’s output (the brake/throttle position) is governed by Eq. (1), where $e_{i}={\Delta v}_{i}=v_{target,i}-v_{i}$ is the error term representing the difference between the set speed and vehicle’s current speed at the current instant of time, *i*.

(1) $$n_{pedal}=K_{P}e_{i}+K_{I}\Delta t\sum_{j=0}^{i}e_{j}+K_{D}\times \frac{e_{i}-e_{i-1}}{\Delta t}$$

*The PID controller for car automation in a discrete simulation environment. j = start time of the controller cycle, i = current time-step in the controller cycle. K_p_ is the proportional gain on the controller. K_i_ is the integral gain of the controller. K_d_ is the gain of the derivative component of the controller*.

The K-coefficients of the sub-controllers represent the controller gains and have a significant impact on the behaviour of the system, as their relative and absolute values determine the rise time, overshoot, and damping characteristics. Therefore, different control states require different settings.

#### Following state

The Follow state aims to maintain a constant time-headway to the lead vehicle. Maintaining headway is a more challenging task than maintaining speed, as the distance is controlled by the host vehicle speed relative to the lead vehicle. The time-headway is set by the driver (in the following increments: 1, 1.5, and 2 s, these values can be changed in the source code in the file ‘OM_Module.cls’ under the ‘cycleTHW’ function) and is defined as $t=d_{lead}/V_{host}$. Additionally, the algorithm will slow down to a stop if there is a crossing vehicle in its path, or if there is a vehicle approaching head on. It will, however, not execute any evasive lateral manoeuvres in its current implementation.

#### Adapt state

The Adapt state is used for smooth speed adjustments to meet the desired target speed. The speed error signal in the Adapt state is defined differently than in other states. The ultimate target speed is still either the speed set by the driver or the externally limited speed (i.e., coming from a slower leading vehicle). However, to attain a smooth manoeuvre and speed adjustment, the error signals refer to instantaneous target speed, which comes from linear interpolation from the vehicle current speed and the target speed.

The interpolated speed is calculated using Bezier curves. Bezier curves are frequently used in computer graphics to render animations or vector graphics. The Bezier curves are used to draw smooth curves that can be scaled dynamically and indefinitely. In animation Bezier curves can be used to control speed over time of animated objects. These characteristics make Bezier functions well suited for use in trajectory planning and interpolation. Bezier functions have been proposed as a way of planning and traversing trajectories in a two-dimensional space by Choi et al. [6]. Such an algorithm would be divided into two parts, trajectory planning and trajectory interpolation [6]. In the current implementation of the control algorithm for longitudinal control the Bezier functions are used to interpolate the speed of the host vehicle to a set speed or a leading vehicle’s speed to ensure smooth acceleration and deceleration by modelling the target speed using a first-order Bezier curve (see Eq. (2)).

(2) $$\left(1-t\right)P_{0}+t\times P_{1}\;,t\in [0,1]$$

*The equation for a first-order Bezier curve. Where P*_*0*_*is the host vehicle speed at the start of the interpolation, and P*_*1*_ *is the target speed. t*_*0*_*is the normalized start point of the interpolation and t*_*1*_ *is the endpoint. The manoeuvre time is calculated and normalized according to Eqs.*(3) *and* (4) *below.*

To plan the speed trajectory, a manoeuvre duration must be computed to match the host vehicle speed with the target speed taking a “comfortable acceleration” threshold as shown in Eq. (3). Following the computation of the manoeuvre duration, the time interval needed is rescaled to a value between 0 and 1 taking the simulator frame rate into account through Eq. (4).

(3) $$T_{manoeuvre}=\frac{{\Delta v}_{i}}{a_{comfortable}}$$

*The formula used for finding manoeuvre duration used for interpolation.*

(4) $$t=\frac{T_{curr}-T_{interp.,start}}{{(T}_{manoeuvre}\times {Hz}_{simulation})}$$

*Rescaling of T_Manoeuvre_ to a scale of 0–1 based on the frequency of the simulator. T*_*curr*_*refers to the current time, T*_*interp., start*_*refers to the start of the interpolation time, T*_*manouvre*_ *refers to the manoeuvre time calculated in Eq.* (3)*.*

When the manoeuvre duration has been determined and scaled to the appropriate range, the current speed, target speed, and time are introduced to Eq. (2) to create the trajectory. Following the creation of the trajectory, the controller set point interpolates along the trajectory until the target speed is reached. This approach ensures that the acceleration threshold is never exceeded.

As the speed is computed at each discrete step of the simulation, the error signals for the PID is significantly smaller than a step input (i.e., from 60 kph to 100 kph), which is more manageable by the PID as the likelihood of an overshoot, or aggressive acceleration is avoided. The error signal for the PID is given by: $e_{i}={\Delta v}_{i}=v_{i}-v_{curr}$ which results in smoother acceleration and deceleration.

#### Cruise state

The cruise state is used when the vehicle does not need to adjust its speed more than 3.5 m/s (i.e., when small adjustments to the throttle output are required to maintain the set speed, when passing through hilly areas or curves), and when there is no lead vehicle or a lead vehicle faster than the set speed. The cruise state controls the speed in accordance with Eq. (1). The error term used for the PID controller is calculated as: $e_{i}=v_{target}-v_{curr}$.

### Lateral automation

The lateral control is responsible for steering the car and controlling its position in the desired lane. This is achieved by controlling the vehicle's lateral position with respect to the road centreline and the centre of the desired lane. The target position is typically the exact coordinate of the centre of the lane with no look-ahead function. Thus, the implemented controller for lateral control is somewhat rudimentary, and other controllers are reported in the literature, as in the work of Hessburg and Tomizuka [7] where a ‘fuzzy’ controller takes in consideration road geometry to steer the vehicle. As STISIM does not afford a look ahead function for roadway geometry, this type of controller was not possible to implement. However, the implemented controller does accommodate for vehicle speed to some extent through a modification of the gain for the steering PID controller.

A vehicle’s trajectory is dependent on both steering angle and vehicle speed. With this in mind, the PID controller was modified to vary the proportional gain of the control signal as a function of current vehicle speed (i.e., a type of gain scheduling) (Eq. (5)). This was also done to compensate for the STISIM vehicle dynamics model that has got some understeer at higher speeds: a higher steering angle must be produced at higher speeds to follow the road’s curvature. The controller is able to keep the vehicle in its lane in most conditions, but in situations where the curve radius is small, and the speed is high, the understeering of the dynamics model causes the vehicle to go out of the lane.

(5) $$n_{steering}={(K}_{1P}+K_{2P}∙{v_{i}}^{K_{3P}}{)\times e}_{i}+K_{I}\Delta t\sum_{j=0}^{i}e_{j}+K_{D}\times \frac{e_{i}-e_{i-1}}{\Delta t}$$

*PID controller for the lateral controller. K*_*1p*_ *is the main proportional scaling factor, K*_*2p*_*is the second scaling factor for the effect of vehicle speed on steering output, V*_*i*_*is the current speed of the host vehicle, e*_*i*_*is the error term (the difference between current lane position and the lane centre). K*_*I*_*is the integral scaling factor, ej is the integrated error term, and K*_*D*_*is the derivative scaling factor.*

### Algorithm performance

A number of tests were carried out to demonstrate the effectiveness of the automated driving toolbox. The test scenario was 10 km long and contained a number of curves and cut-in situations. This scenario was extensively used in Eriksson and Stanton [8] and produces the same vehicle behaviour on repeated tests. The tests are detailed in the sections below.

#### Car following

To assess longitudinal driving performance, the algorithms were run through a motorway driving scenario where a number of cars moved into the host vehicle’s lane, as well as cut-ins as part of double lane changes.

**Fig. 3:**
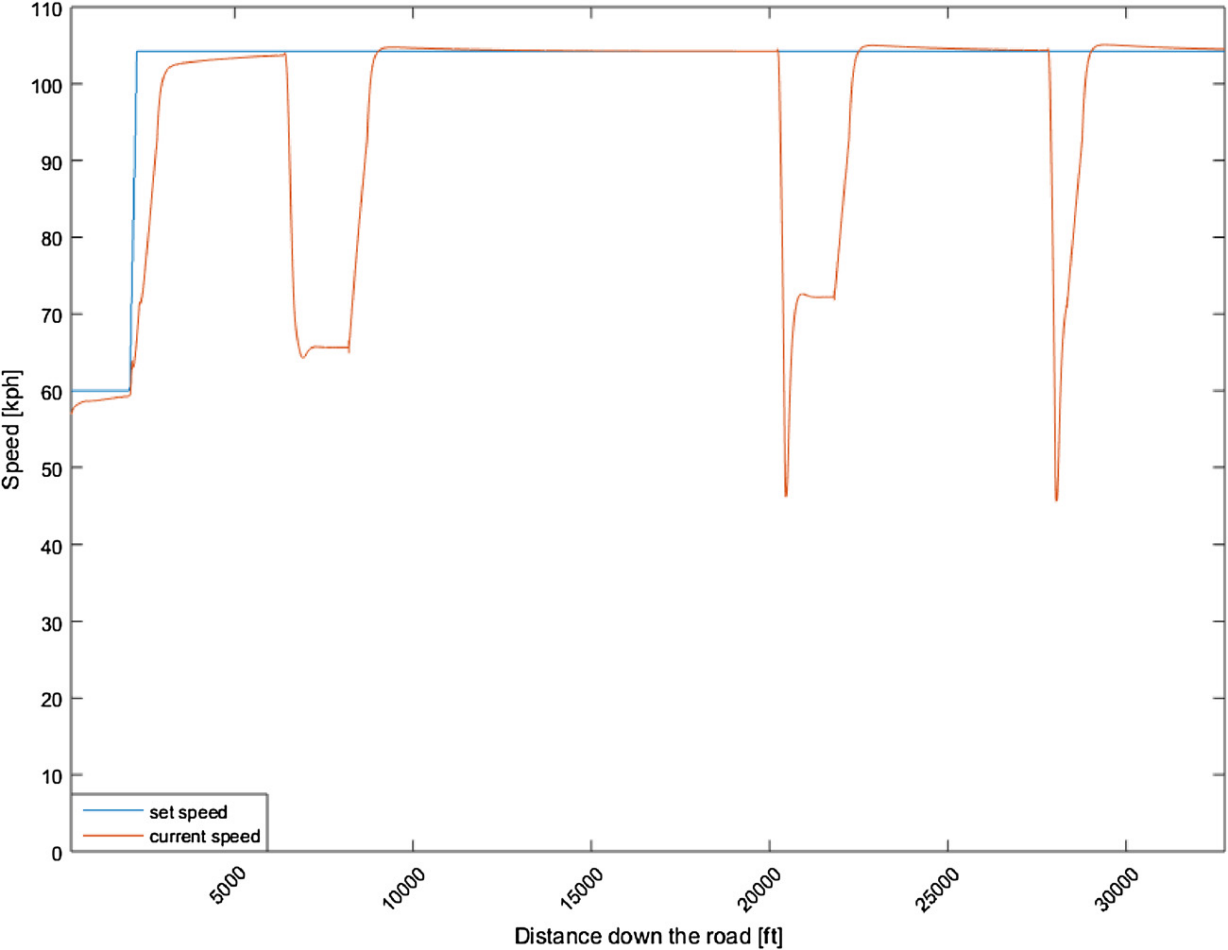
Speed profile of a motorway drive during car following.

**Fig. 4:**
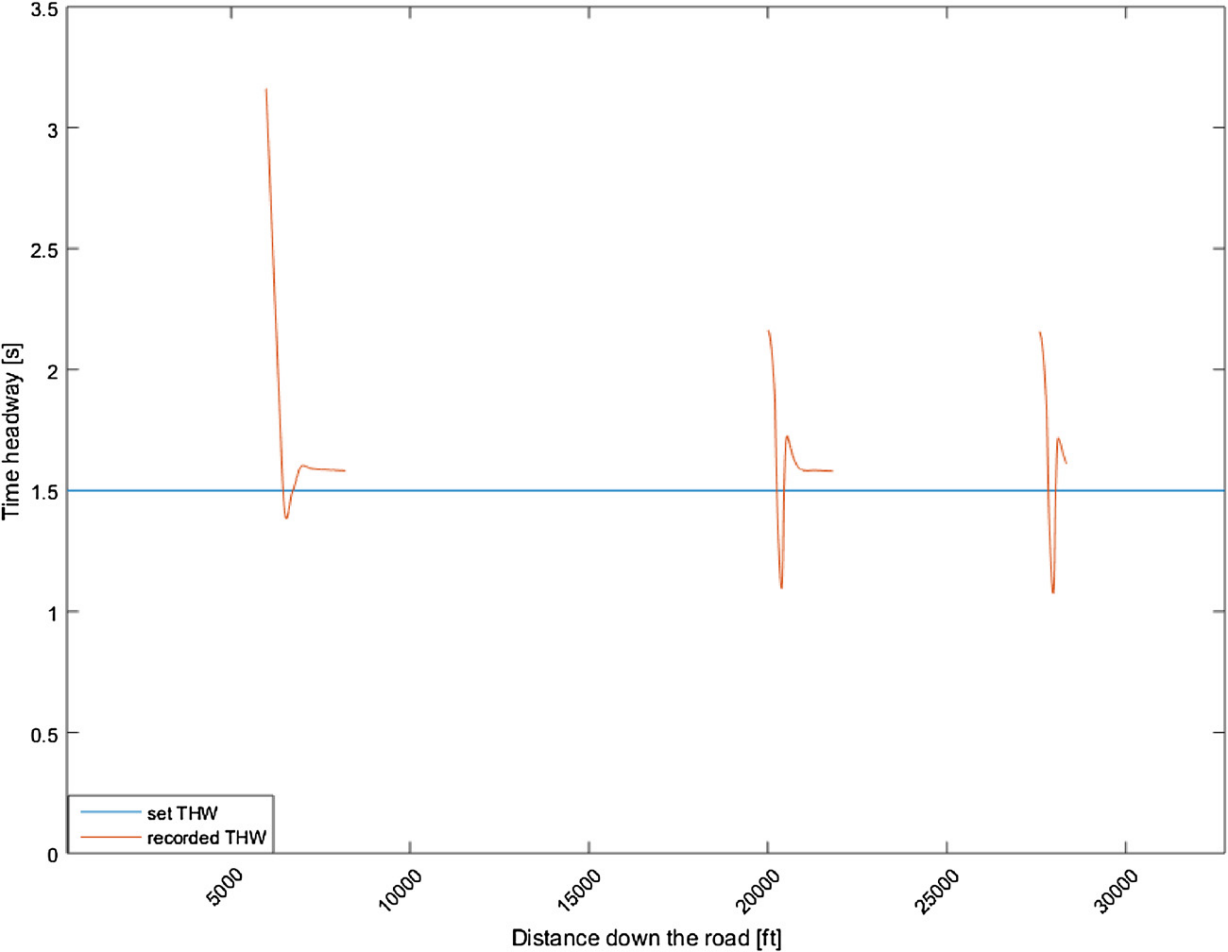
Time-headway profile of the car-following behaviour during motorway driving. The gaps in the recorded time-headway signal are caused by the lead vehicle leaving the host-vehicle lane. THW = time-headway.

Fig. 3 shows the speed profile of the host vehicle in relation to the set speed, whereas Fig. 4 shows the time-headway of any vehicles in front, in relation to the set time-headway (1.5 s). As Fig. 3 shows, the host vehicle slows down below the lead-vehicle speed to accommodate the large need for sudden deceleration to achieve the desired time-headway when there is a large difference in speed between the host and the lead-vehicle caused by the sudden cut-ins. As shown in Fig. 4, the host vehicle closes the gap between the lead and host vehicle down to the desired time-headway and then maintains the desired time-headway consistently. When the lead-vehicle is no longer detected the vehicle then returns to the original set speed.

#### Lane keeping

**Fig. 5:**
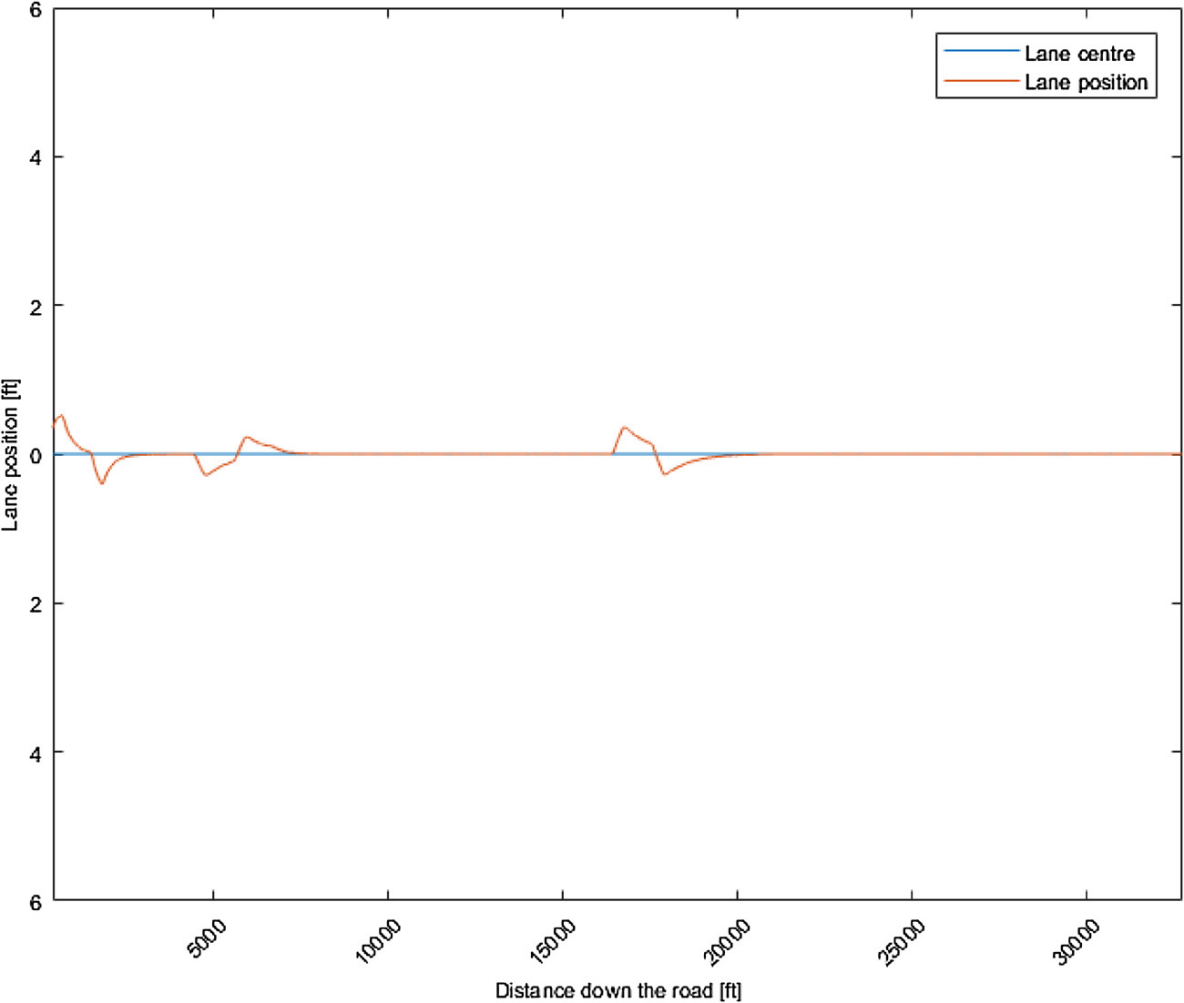
Lane keeping performance (12 ft lane width) during a motorway drive. Lane position refers to the vehicle lateral position in its current lane.

The same motorway scenario was used to assess the automated lateral control of the algorithm. Fig. 5 shows the lateral deviation from the lane centre. It is possible to identify where the vehicle encountered a turn based on the deviation data. However, the lateral deviation is at most ∼15 cm from vehicle centre to lane centre, indicating good lateral vehicle control.

#### Scenario

The following section contains the scenario parameters required to reproduce the drive used in the assessment sections above.

**Figure:**
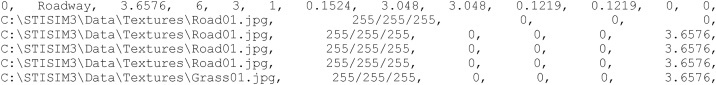



#### Behavioural validity

The software toolbox presented in this manuscript has already been used in research into automated driving with a STISIM driving simulator [8,9]. Eriksson and Stanton [8] assessed the process of driver transitions between automated and manual vehicle control in non-urgent scenarios (SAE Level 4 Automation [10]). To validate the findings from Eriksson and Stanton [8], an on-road study was designed with a matched sample to assess the correlation of the time it took drivers to transition between automated and manual control in the simulator and on the road. The results showed that drivers’ average transitions times from automated to manual control, and vice versa, were about 30% faster for on-road driving than for simulator-based driving; however, the shape of the distributions of transition times was highly similar between simulator-based and on-road driving [11]. The study by Eriksson et al. [11] concluded that there was an indication of relative behavioural validity when the algorithms presented in this manuscript and used in Eriksson and Stanton’s [8] simulator study were contrasted with on-road driving behaviour in a vehicle offering contemporary automated driving.

These findings show that the simulator produces results corresponding to that of on-road conditions. Consequentially, it lends preliminary validity to the use of the algorithms presented in this manuscript for use in research into automated vehicles being conducted in simulators.

#### Limitations

The algorithm outlined above has some limitations when it comes to interacting with certain road environments and road users. The vehicle is unable to navigate intersections (requiring turning), and roundabouts whilst in automated driving mode. Additionally, the software, in its current form, is unable to account for vulnerable road users such as cyclists, pedestrians, and motorcycles. This functionality may be created by accounting for these road users in the lateral and longitudinal control algorithms, should the need arise. As our software toolbox was originally intended for research on automotive automation on non-urban roadways, cyclists, pedestrians, and motorcycles were not implemented.

## How to use the toolbox

The toolbox is able to run out-of-the-box with little set-up and full access to its source code (https://github.com/he1y13/Toolbox-for-automated-driving-in-STISIM) where researchers can make edits and recommit them to GitHub for use by other researchers. The subsections below describe how to set up the toolbox to be run from the pre-compiled Dynamic Link Library (DLL) file and source code.

### Using the pre-compiled DLL file

To use the toolbox using the pre-compiled DLL file, a number of steps must be followed.

1 Create a folder on the C:/ drive of the computer that runs STISIM and name it STISIM (full path of folder: C:/STISIM/)
2 Move the “OM_Automation.dll”, the “ButtonAssignment.txt” and the “shutdownevents.txt” files to C:/STISIM/
3 Open the start menu on the computer, and type in ‘cmd’, right click on the shortcut and click run as administrator
  a Enter the command: cd C:/Windows/SysWOW64/
  b Enter the command: regsvr32 C:/STISIM/OM_Automation.dll
4 Open STISIM to edit the configuration file
  a Open the tab “Data Collection” and tick the box “Collect time to collision data”
  b Open the tab “Open Module” and add the following path to the “Open Module DLL file” box: C:/STISIM/OM_Automation.dll
  c Open the tab “Simulation Control” and change the desired frame rate to 20 frames per second (this value can be edited in the source code to higher values)
5 Open C:/STISIM3/Tools/CalPot32.exe and select the controller being used
  a Open the tab “Test Controls” and click Driver Inputs
  b Open the file C:/STISIM/ButtonAssignment.txt and map the buttons on the controller being used with the corresponding functionality shown in Table 1

**Table 1:** Function mapping in the ButtonAssignment.txt.

Line in file	Function associated with button value
1	Cycle time-headway
2	Increase ACC speed
3	Decrease ACC Speed
4	Activate Adaptive Cruise Control
5	Activate Highly Automated Driving
6	Deactivate automated driving
7	Left lane change
8	Right lane change

When the above steps have been completed, the user may start any scenario (it must be noted that the automation cannot handle intersections and roundabouts) and press the activate button on the controller designated in the “ButtonAssignment.txt” file. Moreover, if the researcher wishes that the automated driving feature should become unavailable at a set point during a drive, this behaviour may be specified in the file “shutdownevents.txt”. This file contains a single event where the researcher may specify a distance down the road (in positive feet down the road, a negative value means it is ignored by the software), the time from the event being triggered to the event occurring (in seconds), the time after the event occurring until the automated driving feature becomes available again (in seconds) in the following manner: “500; 5; 25″ (the event countdown occurs 500 feet down the road counting down for 5 s after which all automated features are unavailable to engage for 25 s).

### Data collection

To save the additional data generated by the toolbox software (such as set time-headway, set speed and level of automation etc.) a number of parameters have been pre-set to be saved into the BSAV data file normally generated in STISIM (the researcher must add variable 49 to be logged as one of the collected parameters in the BSAV event in the scenario definition language). The parameters saved to the data output file are shown in Table 2.

**Table 2:** Parameters from the toolbox collected for data logging purposes saved in parameter 49 of the BSAV event. The units reported in the table are in the units supplied in the Open Module variables.

Parameter	Type of data	Units
1	Distance down the road	feet
2	Level of automation (manual / ACC / Highly automated)	integer (0,1,2)
3	Adaptive Cruise Control state (Cruise, Follow, Adapt)	integer (1,2,3)
4	Desired time-headway	seconds
5	Current time-headway	seconds
6	Desired speed	ft/s
7	Current speed	ft/s
8	Current time to collision	seconds
9	Optimal lane position	feet
10	Current lane position	feet

### Socket connection

The toolbox also allows sending data to an external device, for example, a human-machine interface. This can only be made available through re-compiling the toolbox’s source code after uncommenting a number of lines in the file ‘Open_module.cls’.

The user needs to uncomment lines 700 and 702 to enable the socket connection, and also specify the desired IP address and port to receive the data packets and lines 305–308 to enable data to be sent over the socket. The data being sent is specified in Table 3:

**Table 3:** Pre-specified data being sent over the socket connection when enabled.

Parameter	Type of data	Units
1	Lateral lane position	feet
2	Current lane	integer
3	Selected time-headway	seconds
4–8	Distance to the following vehicles: – lead vehicle in host lane – trailing vehicle right of host lane – leading vehicle right of host lane – trailing vehicle left of host lane – leading vehicle right of host lane	feet
9	Number of lanes on current section of road	Integer
10	Vehicle speed	Ft/s
11	Engine RPM	RPM
12	Automation mode	Integer (0,1,2)
13	Take-over request countdown	Integer (−1 – X)
14	Distance down the road	feet

### Compiling from source code

There are a few additional steps required to run the automation toolbox when edits need to be made in the source code (examples of this would be to add data-output over TCP/IP or to re-tune some of the controllers for longitudinal or lateral control). To make edits to the source code a computer with the Microsoft Visual Basic 6.0 editor installed must be used. When edits to the source code have been made, the new DLL must be compiled; this is done through the drop-down menu “File>Make OM_Automation.dll” in the Visual Basic 6.0 editor.

## Summary

In this manuscript, we described a generic set of algorithms for automated driving research implementable on any simulation platform that allows access to internal variables relating to surrounding traffic through an API/SDK. We then described an implementation of these algorithms on the STISIM driving simulator platform for STISIM V3.07.04 with accompanying performance metrics and a description of validation work. We then provided a step by step guide on how to set up STISIM to access this toolbox for use in research using the Open Source release version of the software. Whilst this implementation is primarily intended for use with STISIM, it is possible to implement the same control functions and finite state machine in other simulators (possibly in a different programming language), provided that they support information acquisition from surrounding traffic in order to provide the controllers with data.

## Additional information

The topic of automated driving receives an increasing level of attention from Human Factors researchers. Until recently, automated driving technology required intermittent driver feedback, for example by touching the steering wheel, thus maintaining a level of driver engagement similar to manual driving [12]. However, recent amendments to the Vienna Convention on Road Traffic enable drivers to be fully hands- and feet-free as long as the system can be overridden or switched off by the driver [13]. This amendment allows drivers to be ‘out-of-the-loop’ for prolonged periods of time, yet drivers are still expected to resume control when the operational limits of the automated driving system are approached [10].

The availability of these highly automated driving systems may fundamentally alter the driving task, and could give rise to ‘ironies’ and ‘surprises’ of automation similar to those proposed by Bainbridge [14] and Sarter et al. [15] in the context of process control and aviation. Indeed, several empirical studies have shown that drivers of highly automated cars often respond slowly when manual intervention is necessary [[16], [17], [18], [19], [20]]. In light of this, intermediate forms of automation have been deemed hazardous because drivers are required to be able to regain control at all times [21,22]. To study these psychological phenomena and develop effective Human-Machine Interfaces for supporting drivers of future automated cars, the driving simulator is seen as a viable option [11,23].

### Simulators

Driving simulators have been used since the beginning of the 1930s [24] and Human Factors research into automated driving has been ongoing since the mid-1990s [25,26]. Compared to on-road testing, driving simulators allow driver reactions to new technology to be measured in a virtual environment, without physical risk [[27], [28], [29], [30], [31]].

Furthermore, driving simulators offer a high degree of controllability and reproducibility, and provide access to variables that are difficult to accurately determine in the real world [32], such as lane position and distance to roadway objects [33,34]. Most driving simulators offer flexibility in designing custom plug-ins through APIs. With Open Source software efforts in driving simulation, such as OpenDS [35], it is likely that the use of driving simulators will come to grow in the coming years.

### STISIM

STISIM is a popular driving simulator that is used for research purposes [[36], [37], [38], [39], [40], [41]]. The STISIM driving simulator software comes with an ‘Automated Driving’ feature accessible through its Scenario Definition Language (SDL) [42,43]. The SDL-based automation allows the researcher to enable or disable automated lateral and/or longitudinal control through the ‘Control Vehicle’ (CV) event by specifying a distance down the road at which point the event should trigger, and what mode change should occur (e.g., the script *‘2000, CV, speed,* 2′ initiates automated control of both steering and speed when the participant has travelled 2000 m along the road). The STISIM documentation states that this automated driving feature is intended for driver training [44], an approach also taken by other driving simulator manufacturers (e.g. [45]). That is, by enabling automated control of speed, the driver can fully concentrate on learning how to steer, or vice versa, by enabling automated control of steering the learner driver can concentrate on how to accelerate and stop the car. This type of automation is sufficient when it comes to research where the researcher does not want the driver to be able to (dis)engage the automation or change the automation modes. The CV event has been successfully used in this manner (as described by [46,47]; and presumedly also in similar studies using STISIM: [40,[48], [49], [50], [51]]). However, if the research aims to understand how drivers *interact* with automated driving systems, as in Kircher et al. [52], Eriksson and Stanton [8] and Eriksson et al. [11], this type of hard-coded automation is not sufficient.
